# Ferroptosis and WDFY4 as novel targets for immunotherapy of lung adenocarcinoma

**DOI:** 10.18632/aging.205042

**Published:** 2023-09-19

**Authors:** Ling Huang, Lifan Zhong, Ruxin Cheng, Limei Chang, Mingyan Qin, Huaping Liang, Zhongkai Liao

**Affiliations:** 1 State Key Laboratory of Trauma, Burns and Combined Injury, Department of Wound Infection and Drug, Daping Hospital, Army Medical University, Chongqing, China; 2 Hainan Center for Drug and Medical Device Evaluation and Service, Hainan Medical Products Administration, Haikou, China; 3 School of Hainan Provincial Drug Safety Evaluation Research Center, Hainan Medical University, Haikou, China; 4 Emergency and Trauma College, Hainan Medical University, Haikou, Hainan, China; 5 Department of Thoracic Surgery, The Second Affiliated Hospital of Hainan Medical University, Haikou, China

**Keywords:** Single Cell RNA-Sequencing, lung adenocarcinoma, WDFY4, immunotherapy, ferroptosis

## Abstract

Background: Lung cancer exhibits the world’s highest mortality rate among malignant cancers worldwide, thereby presenting a significant global challenge in terms of reducing patient mortality. In the field of oncology, targeted immunotherapy has emerged as a novel therapeutic approach for lung cancer. This study aims to explore potential targets for immunotherapy in lung adenocarcinoma (LUAD) through the analysis of Ferroptosis Index (FPI) and Single Cell RNA-Sequencing (scRNA-seq) data. The findings of this research can potentially offer valuable insights for improving LUAD immunotherapy strategies and informing clinical decision-making.

Methods: Firstly, the relationship between survival and ferroptosis in LUAD patients was analyzed by FPI. Subsequently, the association between ferroptosis and infiltration and regulation of immune cells was explored by immune infiltration analysis and correlation statistics. Lastly, the relationship between major infiltrating immune cell populations and related pathways and prognosis of LUAD patients was analyzed by GSEA and GSVA. To screen out core genes regulating infiltration of immune cell populations, scRNA-seq data of cancer and para-cancerous tissues of LUAD patients were downloaded, followed by cell clustering analysis, cell identification of core subpopulations, pseudotime analysis, single-cell GSVA and pathway enrichment analysis, and identification and functional analysis of core regulatory genes. Moreover, the expression levels of core functional genes in LUAD tissue microarray were detected by immunohistochemistry, and its relationship with the prognosis of LUAD patients was verified. Finally, we used lentivirus with WDFY4 to transfect LUAD A549 cells. CCK-8, flow cytometry apoptosis detection, Scratch wound healing assay, Transwell migration assay, Xenograft nude mice model, immunohistochemical analysis and other experimental methods were used to explore the biological effects of WDFY4 on LUAD *in vitro* and *in vivo*.

Results: Survival analysis of FPI values in LUAD patients revealed a positive correlation between smaller FPI values and longer overall survival. Immuno-infiltration analysis and its correlation with FPI values revealed that B cells were most strongly associated with ferroptosis. Ferroptosis of cancer cells could promote infiltration and activation of B cell populations, and LUAD patients with more infiltration of B cell populations had longer long-term survival. scRNA-seq data analysis indicated that the B cell population is one of the major cell populations infiltrated by immune cells in LUAD. During the later phases of B cell differentiation in LUAD, there was a decrease in the expression levels of ACAP1, LINC00926, TLR10, MS4A1, WDFY4, and TRIM22 genes, whereas the expression levels of TMEM59, TP53INP1, and METTL7A genes were elevated. The protein-protein interaction (PPI) network analysis indicated that WDFY4 plays a crucial role in regulating B cell differentiation in LUAD. Immunohistochemical analysis of LUAD tissue microarray revealed a significant downregulation of WDFY4 expression, which was closely related to the occurrence sites of LUAD. Moreover, LUAD patients with a low WDFY4 expression exhibited a poorer prognosis. Additionally, experimental findings demonstrated that the overexpression of WDFY4 could inhibit the proliferation and metastasis of A549 cells while promoting apoptosis. It was also confirmed that WDFY4 could inhibit cancer growth *in vivo*.

Conclusions: The results indicate that promoting infiltration and activation of B cell populations could improve the long-term survival of LUAD patients, thereby offering a potential novel immunotherapeutic approach for LUAD. Besides, the promotion of cancer cell ferroptosis and upregulation of WDFY4 expression have been shown to induce the infiltration and activation of B cell populations. Furthermore, the overexpression of WDFY4 can significantly inhibit the growth of lung adenocarcinoma *in vitro* and *in vivo*, highlighting its potential as a target for immunotherapy in LUAD.

## INTRODUCTION

Lung cancer is the most prevalent malignant cancer and the leading cause of cancer death. It was reported that there were about 2.2 million new cases and 1.79 million deaths of lung cancer in 2020, accounting for 22.4% and 18.0% of total cancer cases, respectively [1], with non-small cell lung cancer (NSCLC) accounting for 85% of the morbidity. In NSCLC, the incidence rate of LUAD has increased in recent years, and it has surpassed lung squamous carcinoma as the most prevalent histological type of lung cancer [2], so the treatment of LUAD has been one of the most critical tasks for treating lung cancer. Currently, surgery is still the main treatment for LUAD, combined with radiotherapy, immunotherapy, and molecular targeted therapy. Despite recent advancements in the treatment of LUAD the 5-year survival rate remains considerably low, standing at approximately 15% [3]. The reasons are that most lung cancer patients are diagnosed at advanced stage when diagnosed, resulting in a low surgical resection rate, with chemotherapy resistance, severe side effects, and insensitivity to molecular targeted therapy [4]. Consequently, effective treatments to improve the success rate of targeted therapy for LUAD are important to prolong the survival time of lung cancer patients.

Cancer immunotherapies, such as immune checkpoint blockade, have dramatically changed the way cancer is treated and improved the long-term survival of many cancer patients, but not all cancers and patients respond to immunotherapy, so specific mechanisms of response or non-response to immunotherapy and corresponding biomarkers are needed to improve the effectiveness of immunotherapy [5]. Tumor-infiltrating B cells have been found in various cancer tissues, exerting influence on tumorigenesis and the efficacy of immunotherapy. Studies have shown a positive correlation between the response to immunotherapy and B cells in cohorts of metastatic melanoma, renal cell carcinoma, and soft tissue sarcoma [6, 7]. Cancer-infiltrating B cells provide a novel perspective on cancer immunotherapy, new biological markers for immunotherapy and guidance for clinical decision-making.

The presence of diverse genomic clones within a cancer poses a significant challenge in the treatment of cancers. This heterogeneity directly impacts the clinical diagnosis and treatment of lung cancer [8, 9]. Single Cell RNA-Sequencing Analysis (scRNA-seq) offers a comprehensive approach to studying the occurrence and progression of lung cancer, thus facilitating the diagnosis and treatment of this disease [10, 11]. For example, some researchers have utilized the scRNA-seq technique to identify core genes such as TREM2, CD81, MARCO, and APOE, which play a crucial role in regulating the infiltration of the cancer microenvironment by macrophages in LUAD [12], They also found that changes in stromal and immune cells in metastatic LUAD promoted an immunosuppressive microenvironment [13]. Moreover, they screened out cell subpopulations associated with drug resistance in LUAD [14]. Therefore, techniques of scRNA-seq assays and analysis are conducive to finding diagnostic marker genes and therapeutic targets for LUAD.

Ferroptosis is a recently discovered form of programmed cell death mediated by iron-dependent accumulation of lipid peroxidation [15]. Several studies have shown that ferroptosis is involved in the occurrence, progression and treatment of malignant cancers and has a dual impact on in cancer immunoregulation, as it induces ferroptosis in cancer cells to mediate anticancer immunity while also promoting immune escape of neighboring cancer cells and cancer growth on the other hand [16, 17]. In the present study, we first demonstrated the role of ferroptosis in the progress of LUAD by Ferroptosis index (FPI) analysis. Besides, we found that inducing ferroptosis could promote B cell cancer infiltration and activation, and survival was prolonged in LUAD patients with B cell infiltration. Furthermore, WDFY family number 4 (WDFY4) was found to be important in regulating B cell infiltration and differentiation using two sets of scRNA-seq data analysis in LUAD tissues and para-cancerous tissues. The analysis of the LUAD tissue microarray revealed a significant decrease in WDFY4 expression within cancerous tissues, which correlated with a shorter overall survival rate among LUAD patients. As a result, our study suggests that it might be a novel strategy for LUAD immunotherapy to promote cancer infiltration and activation of B cells by inducing ferroptosis in cancer cells and elevating WDFY4 expression.

## MATERIALS AND METHODS

### FPI identification and sample grouping

In accordance with the findings of Liu et al. [18], a total of 24 ferroptosis regulator genes (FRGs) were identified and categorized into two groups based on their respective modes of action: positive and negative genes. The positive regulation of gene expression was observed through FPI’s influence on LPCAT3, NCOA4, ACSL4, GPX4, SLC3A2, ALOX15, SLC7A11, NOX5, NFE2L2, NOX3, NOX1, and NOX4. Conversely, the negative regulation of gene expression was evident in the case of FDFT1, COQ10A, HMGCR, and COQ10B, among others. The FPI value was determined by calculating the enrichment scores (ES) of positive regulatory genes minus the ES of negative regulatory genes by the single-sample gene-set enrichment analysis (ssGSEA) algorithm. Subsequently, a survival analysis was conducted on the FPI values in LUAD patients using the x-title algorithm. The results indicated that an FPI < 0.47 is associated with improved survival in LUAD patients, whereas patients with an FPI > 0.47 exhibit a poorer long-term prognosis. The samples were subsequently categorized into high- and low-score groups based on their PFI scores. Variance analysis was conducted using LIMMA, and the differential genes were determined based on the following criteria: log2 fold change ≥1.5 and Benjamin-Hochberg (B-H) adjusted *P*-value < 0.05.

### Immune infiltration and correlation analysis

Studies have confirmed that FPI is closely related to immune regulation, although the precise regulatory mechanism in LUAD remains unknown. In order to investigate this potential relationship further, we utilized ssGSEA to calculate the relative degree of cellular infiltration for each subtype, based on the expression levels of 24 immune cell surface markers as outlined by Finotello et al. [19]. Additionally, we conducted bias correlation analysis to examine the relationship between the relative infiltration values and PFI values of each cell subtype of cells, following standard quantification. The resulting correlation statistics were adjusted by Pearson’s method.

### Gene set enrichment analysis (GSEA)

The gene-set-based enrichment analysis method known as GSEA is employed in the analysis of gene expression data. Initially, the purpose is established, followed by the selection of gene sets from MSigDB are selected for systematic analysis. The gene expression data are then ranked based on their association with the utilizing normalized scores. Subsequently, the evaluation of the impact of synergistic changes in genes within the gene sets on phenotypic changes is conducted by determining if the genes within each gene set are enriched for the clinical phenotypic correlation. The calculated parameters for this analysis were as follows: minGSSize = 10, maxGSSize = 500, and B-H adjusted *p*-value Cutoff = 0.05.

### Gene set variation analysis (GSVA) and survival analysis

Gene Set Variation Analysis (GSVA) is a non-parametric, unsupervised technique that distinguishes itself from Gene Set Enrichment Analysis (GSEA) by its ability to compute enrichment scores for individual gene sets in each sample without the need for pre-grouping of samples. In other words, GSVA transforms a gene sample matrix into a gene-set by sample matrix, facilitating the assessment of pathway enrichment for each sample. This approach offers a standardized quantification of gene enrichment results, thereby enabling statistical analysis of differences in subsequent pathway sets. Following that, by employing the LIMMA package for differential expression analysis, which relies on GSVA scores, it becomes feasible to discern genes that exhibit differentially expression across samples. The resultant sets of differentially expressed genes possess greater biological significance and are more amenable to interpretation in the context of disease progression. Moreover, leveraging GSVA scores, an additional prognostic analysis was performed using K-M survival analysis to elucidate the influence of pathways on the long-term survival of patients.

### Single-cell RNA clustering

Based on the single-cell data, we first performed quality control on the downloaded raw data to filter out interfering impurities. The criteria employed for this purpose encompassed cellular gene count, mRNA count, ribosomal genes, etc. The preprocessing parameters utilized were as follows: min Gene = 500, max Gene = 4000, and the percentage of mitochondrial genes <5%. Subsequently, principal components analysis (PCA) was employed to downscale highly variable genes, and the resulting specific components analysis (PC) values were further downscaled to a two-dimensional space using linear downscaling techniques such as t-distributed stochastic neighbor embedding (tSNE) or uniform manifold approximation and projection (UMAP), following the selection of an appropriate PCA dimension. After the aforementioned procedure, the majority of feature values in the transcriptional profile were represented in a two-dimensional format. Prior to conducting PCA downscaling, the expression data underwent centering, and cell cycle regression analysis was performed using the Scale Data function.

### Cell identification of core subpopulations, pseudotime analysis, and variance analysis

To identify the significantly highly expressed genes within each cluster, we used the Find All Markers function from the Seurat package. This function allowed us to obtain differential genes by configuring the test use parameter and conducting Wilcox differential statistics analysis, with *p*-value < 0.05 to define differential genes. Following the completion of cell clustering and downscaled visualization we were able to demonstrate the heterogeneity of the cell population. To annotate the cell subpopulations within each cluster, we used the Single R and scCATCH algorithms on the differential genes obtained from each cluster. To elucidate the developmental status and potential regulatory nodes of each subpopulation, we conducted pseudotime analysis on the transcriptional profiles of cell subpopulations based on the Monole algorithm. Subsequently, we examined the pseudotime genes that are relevant to development.

### Single cell GSVA and pathway enrichment analysis

We estimated the kernel density of the pathway sets for the transcriptional profile level in a single cell based on the unsupervised GSVA algorithm, which shares similarities with the nonparametric test method used in probability theory to estimate the unknown density functions. Gene sets were compiled based on specific gene sets of interest, such as signaling pathways and GO entries. The RNA-seq count data of genes in each cell were calculated iteratively on the gene expression matrix, allowing for the exploration of enrichment values for specific pathways in each cell. Based on the grouping, cell status and subpopulation information, variance analysis was performed on the GSVA values. The method was the same as described in Method 4.

### Identification and functional analysis of core regulatory genes

To probe the potential role of ferroptosis genes in regulating core cell populations during the progression of LUAD, we conducted an analysis that involved the intersection of single-cell pseudotime genes and ferroptosis-related genes. Subsequently, we elucidated the functional changes of these genes using a pseudotime heat map. Additionally, for the core genes, we employed protein-protein interaction (PPI) network analysis to gain further insights into their protein function and interactions. To accomplish this, we initially utilized the String online database (https://www.string-db.org/) was used to analyze the network, followed by network contribution analysis using Cytoscape. The primary methods employed for the interactions included Textmining, Experiments, Databases, Co-expression, Neighborhood, Gene Fusion, and Co-occurrence, with an interaction score threshold of 0.4. Additionally, the biological functions and protein substructure binding thresholds were analyzed using the Toppgene database (https://toppgene.cchmc.org), and the drug targets of core genes by CMAP database. A significance level of B-H adjusted *P*-value < 0.05 was deemed indicative of a significant difference.

### Cell lines and cell culture

Human lung adenocarcinoma A549 cells were obtained from the Cell Bank of the Chinese Academy of Sciences (Shanghai, China) and were cultured in Roswell Park Memorial Institute 1640 (RPMI 1640) with 10% fetal bovine serum (FBS; Gibco Life Technologies, NY, USA), and 1% penicillin–streptomycin (Gibco Life Technologies, NY, US) at 37°C in a humidified incubator with 5% CO_2_.

### Establishment of WDFY4 overexpressing cell line

The lentivirus carrying the WDFY4 gene sequence was obtained from Hanbio Co. LTD (Shanghai, China). Lung adenocarcinoma A549 cells were seeded in 12-well plates and then infected with lentiviruses following the manufacturer’s recommended protocol. After 24 hours, serum-free medium was replaced with complete medium. To establish a stable cell line with overexpressed WDFY4, lentivirus-infected cells were chosen by incubation with 2 μg/ml puromycin.

### Cell counting Kit-8 assay

The 2000 A549 cells transfected with lentivirus (ad-WDFY4) were inoculated in a 96-well plate and cultured in an incubator for a predetermined duration (24, 48, 72 h). After incubation with CCK-8 solution (Beyotime Biotechnology, Shanghai, China) for 2 h, the absorbance (450 nm) was measured by a microplate reader.

### Cell apoptosis assay

Ad-WDFY4/A549 cells were cultured for 24 h, followed by digestion with 0.25% trypsin and subsequent harvesting. Subsequently, Annexin V-PE/propidium iodide (PI) apoptosis detection kit (Beyotime Biotechnology, Shanghai, China) was used to evaluate the degree of apoptosis by flow cytometry. The percentage of apoptotic cells was calculated as the sum of the early and late apoptotic cells located in the lower right quadrant and upper right quadrant, respectively.

### Scratch wound healing assay

The cells were seeded in 6-well plates and cultured until reaching approximately 80% confluence. A scratch was made in the middle of the culture dish using a 10 μL pipette tip. Photographs were captured immediately after the scratch was made and again after 24 hours. Image J software was used to analyze the migration distance and calculate the migration rate.

### Transwell migration assay

The transfected A549 cells were seeded in the upper chamber of a serum-free medium (filter membrane 8 μm). Complete medium containing 10% FBS was added to the lower chamber. After a 24-hour incubation period, the upper chamber was taken out and fixed in a 4% PFA solution at room temperature for 10 min. Subsequently, the cells were stained with a 0.1% crystal violet solution and imaged.

### Western blotting

Cells were harvested and lysed in RIPA buffer (Beyotime Biotechnology, Shanghai, China), Subsequently, the protein concentration was measured using the BCA kit (Beyotime Biotechnology, Shanghai, China). An equal amount of cell lysate was separated by SDS-PAGE and transferred onto a PVDF membrane. The PVDF membranes were then blocked with 5% skimmed milk and incubated overnight at 4°C with primary antibodies against WDFY4 (1:1000, Abcam, Waltham, MA, USA). This was followed by incubation with polyclonal HRP-labeled secondary antibodies (1:5000, MultiSciences, Shanghai, China) at room temperature for 1 h. Finally, the membrane was developed using enhanced chemiluminescence.

### Xenograft nude mice model

All animal experiments conducted in this study were granted approval by the Institutional Animal Care and Use Committee of Research Center for Drug Safety Evaluation of Hainan Province, Hainan Medical University (2023-0320 (NM01)) and were performed in strict adherence to the established guidelines. BALB/c nude male mice (4–5 weeks old) were provided by the Beijing Vital River Laboratory Animal Technology Co., Ltd. A total of 1 × 10^7^ A549 cells, transfected with control or WDFY4 lentivirus, were subcutaneously injected into the left dorsal region of nude mice. Tumor volume and weight were evaluated at 2-day intervals. Tumor volume was calculated according to the following formula: length × width2/2 (mm3). On the 15th day, the mice were euthanized via cervical dislocation following anesthesia with pentobarbital sodium. Tumor tissue blocks were collected for subsequent immunohistochemical staining analysis.

### Immunohistochemical analysis

The IHC assays and IHC scores were performed according to a previously published protocol [20, 21]. High expression levels were defined as a cumulative score of 4 points, while low expression levels were determined by a cumulative score of less than 4 points. The LUAD tissue microarray (HLugA180Su08, XT19-019) was obtained from Shanghai Outdo Biotech, including 98 cases of LUAD tissues and 82 cases of adjacent tissues. The primary antibodies used, including WDFY4, Ki-67, and Caspase-3, were purchased from Abcam (1:200, Abcam, Waltham, MA, USA).

### Statistical analysis

The statistical analysis was performed using GraphPad Prism software 8.0 (GraphPad Software, Inc., San Diego, CA, USA). The data were represented as means ± standard deviations. For the cell and animal experimental data, statistical significance was determined using the student’s *t*-test or one-way ANOVA. The expression level of WDFY4 in lung cancer tissues and noncancer tissues was analyzed by Student’s *t*-test. Chi-square test was used to analyze clinicopathological features, and Kaplan-Meier method and log-rank test were applied for survival analysis. *P* < 0.05 was considered statistically significant [22].

### Data availability statement

The original contributions presented in this study are included in this article/Supplementary material, the research and analysis data are from the GEO database, and further inquiries can be directed to the corresponding authors.

## RESULTS

### FPI identification and sample grouping analysis

**Figure 1:**
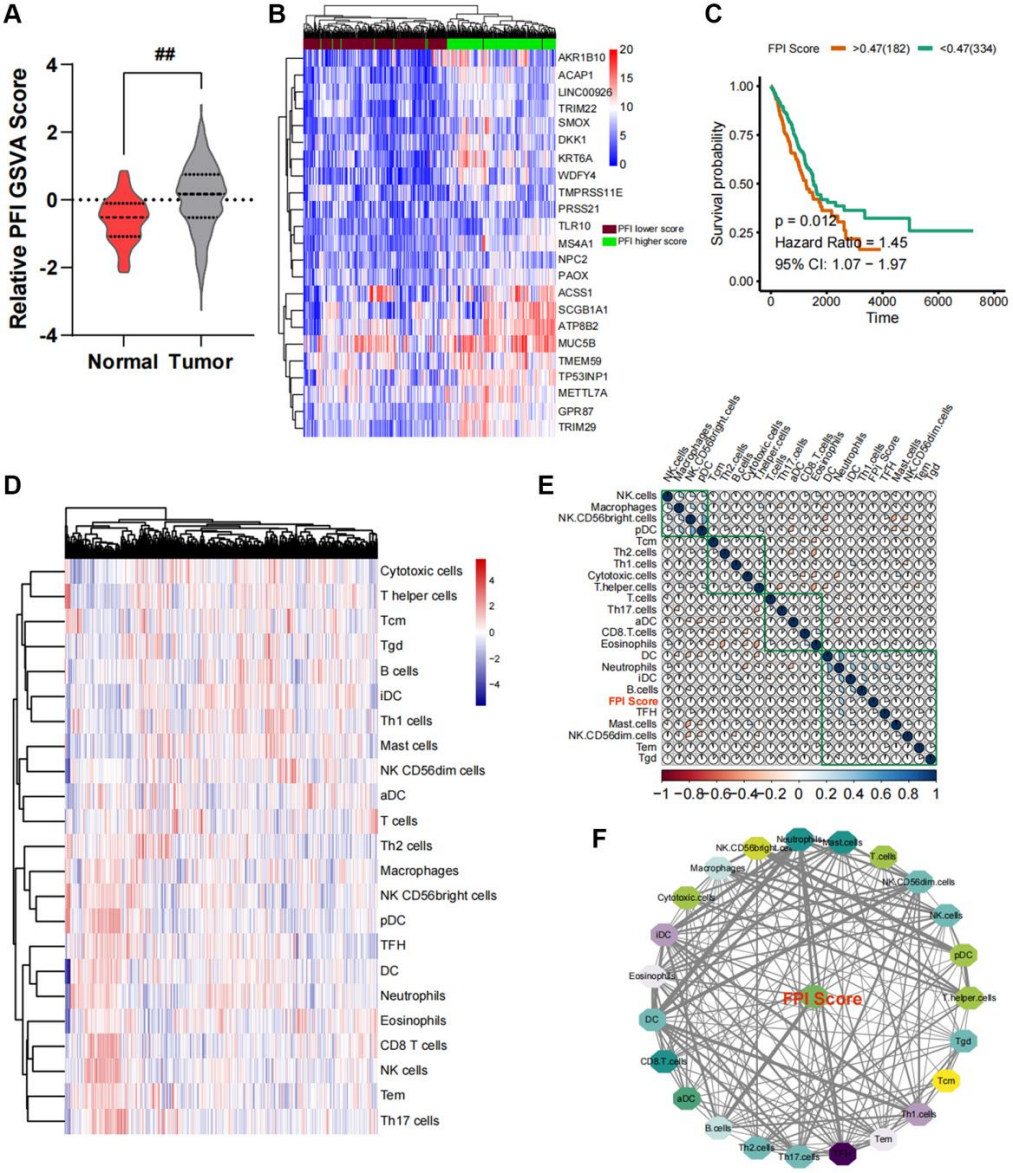
**Construction of an interaction network between the ferroptosis index (FPI) and the cancer immune microenvironment.** (**A**) Variance analysis of standardized FPI between normal and cancer tissues in lung cancer. (**B**) Changes in the expression profile of ferroptosis-related genes. (**C**) Survival analysis and post-analysis of standardized FPI. (**D**) Analysis of immune cell infiltration in lung cancer tissues and relative expression abundance. (**E**) Correlation heat map of relative infiltration values of local immune cells and FPI. (**F**) Analysis of strongly associated immune cell subpopulations by Pearson correlation, with FPI as the core of regulation.

Each LUAD sample in the TCGA had its FPI quantified based on the GSAV analysis. As shown in Figure 1A, the FPI values in LUAD cancer tissues were lower than those in para-cancerous tissues The FPI values of all TCGA-LUAD cancer samples were then analyzed for survival. Utilizing the survival and survminer algorithms, the optimal cutoff value was determined to establish a correlation between FPI and survival in the samples. This cutoff value was then used to classify the samples into FPI high- and low-expression groups. Figure 1B illustrates the differential genetic profile of the two sample groups. The lower the FPI value, the shorter the long-term survival rate of LUAD patients (Figure 1C).

### Immuno-infiltration analysis and correlation statistics

**Figure 2:**
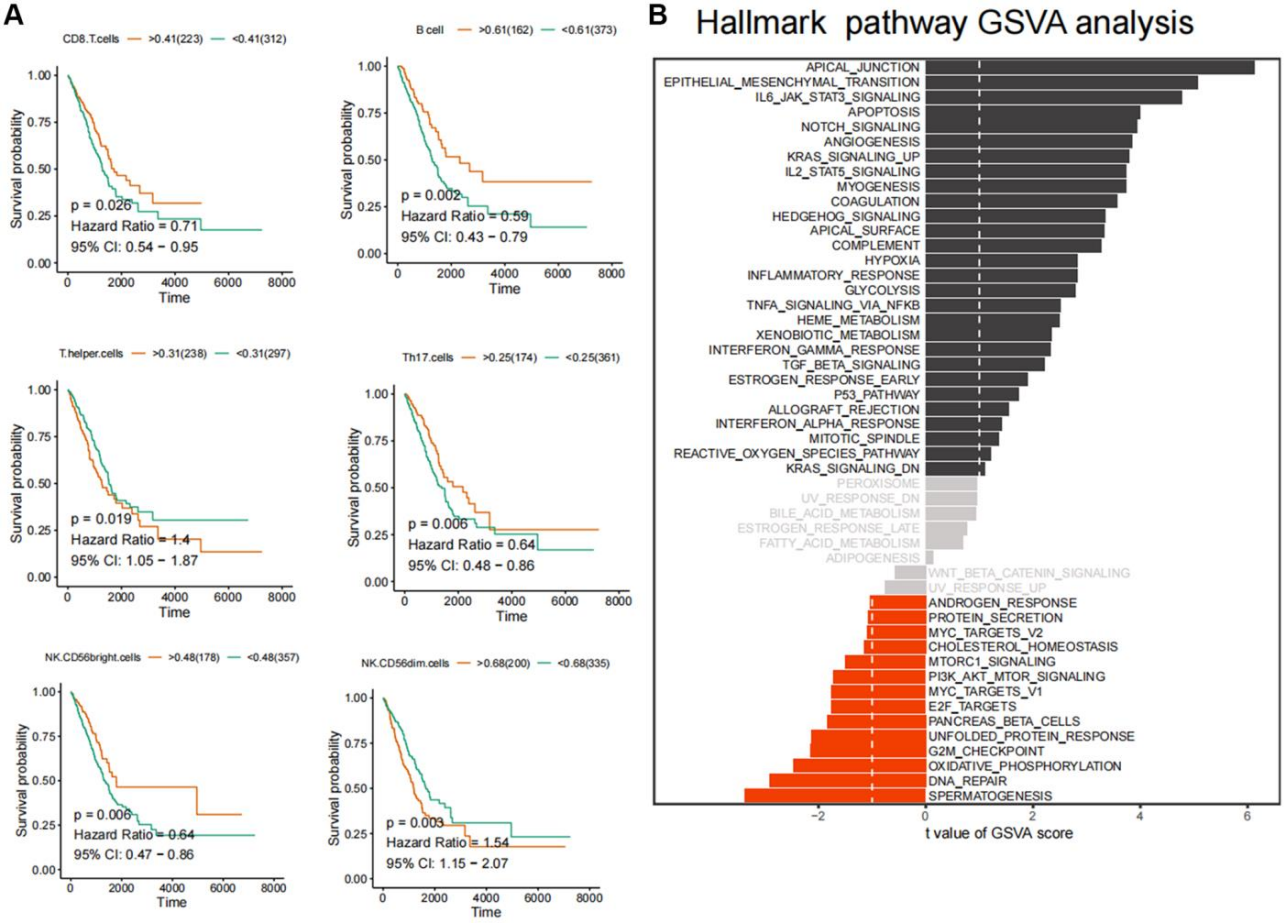
**Identification of core immune subpopulations and variability analysis of GSVA pathway.** (**A**) Correlation analysis between ferroptosis-related immune cell populations and survival prognosis of lung cancer patients. (**B**) Variability analysis of the Hallmark pathways in cancer and normal tissues.

In order to quantify the relative infiltration of each cell within the LUAD cancer samples, an immuno-infiltration analysis was conducted using ssGSEA, which involved the assessment of 24 immune cell genes as markers (Figure 1D). Through partial correlation analysis, the correlation between FPI values and the standard relative infiltration values of immune cells was determined. The application of automated clustering techniques resulted in the identification of immune cell populations associated with FPI (Figure 1E). The analysis of the interaction network between FPI values and immune cells indicated a significant correlation between the infiltration levels of B cells, CD8 T cells, T helper cells, Th17 cells, NK CD56bright cells, and NK CD56dim cells with ferroptosis (Figure 1F). Among these, B cells exhibited the strongest association with ferroptosis (Figure 1F). Furthermore, the above-mentioned immune cell infiltration was closely related to the long-term survival prognosis (Figure 2A).

### GSVA and survival analysis

**Figure 3:**
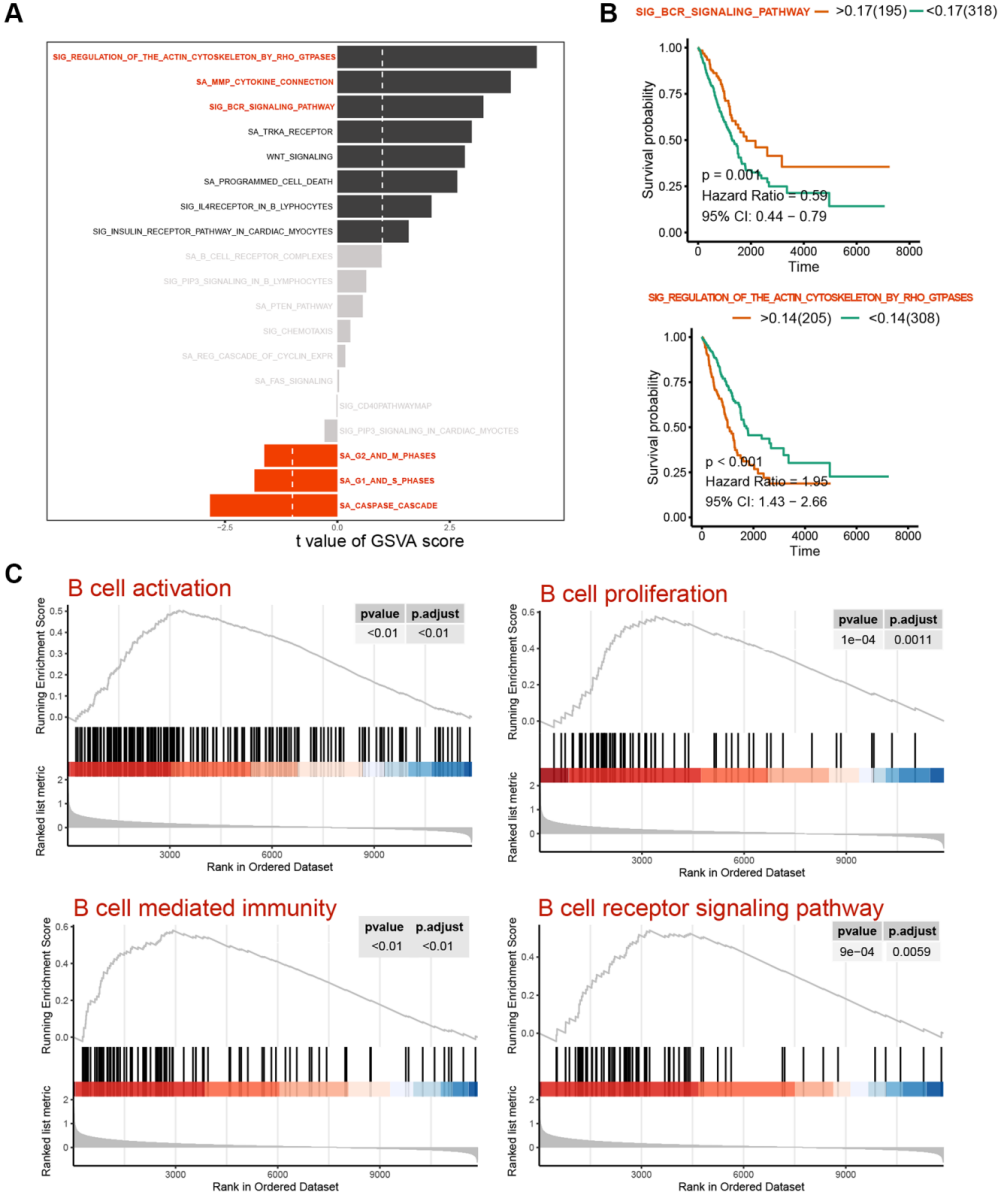
**Variability analysis of immunoregulation-related pathways.** (**A**) Differential analysis of variability scores of immunoregulation-related pathways in GSEA. (**B**) Survival analysis of the SIG_BCR_SIGNALING_PATHWAY and SIG_REGULATION_OF_THE_ACTIN_CYTOSKELETON_BY_RHO_GTPASES in patients with LUAD. (**C**) Enrichment significance analysis of B Cell activation and immunoregulatory pathways.

Subsequently, we conducted GSVA pathway quantification on genes belonging to the HALLMARKER gene set. According to the B-H difference calculation in the LIMMA algorithm, we observed significant upregulation in the expression levels of apical junction, epithelial mesenchymal transition, IL6/JAK/STAT3 signaling, apoptosis and Notch signaling pathways. Conversely, the expression levels of unfolded protein response, G2M checkpoint, oxidative phosphorylation, DNA repair, spermatogenesis and other pathways were significantly downregulated (Figure 2B). Furthermore, we conducted GSVA standard quantification and differential analysis on the immune gene sets. The results indicated that SIG_REGULATION_OF_THE_ACTIN_CYTOSKELETON_BY_RHO_GTPASES, SA_MMP_CYTOKINE_CONNECTION and SIG_BCR_SIGNALING_PATHWAY showed significant up-regulation. Moreover, SIG_REGULATION_OF_THE_ACTIN_CYTOSKELETON_BY_RHO_GTPASES and SIG_BCR_SIGNALING_PATHWAY was strongly associated with the long-term survival of LUAD patients (Figure 3A, 3B). In this case, patients with higher GSVA values of SIG_BCR_SIGNALING_PATHWAY had longer overall survival, whereas the opposite trend was observed for SIG_REGULATION_OF_THE_ACTIN_CYTOSKELETON_BY_RHO_GTPASES (Figure 3B).

### GSEA analysis

The GSEA flow analysis revealed that the PFI low-score group exhibited elevated expression levels of pathways of B cell activation, B cell proliferation, B cell-mediated immunity and B cell receptor signaling pathway were high in the PFI low-score group, whereas the PFI high-score group displayed lower expression levels of these pathways (Figure 3C). These findings suggest a strong association between ferroptosis and B-cell activation, both of which are closely linked to the long-term survival of patients.

### ScRNA cell clustering analysis

**Figure 4:**
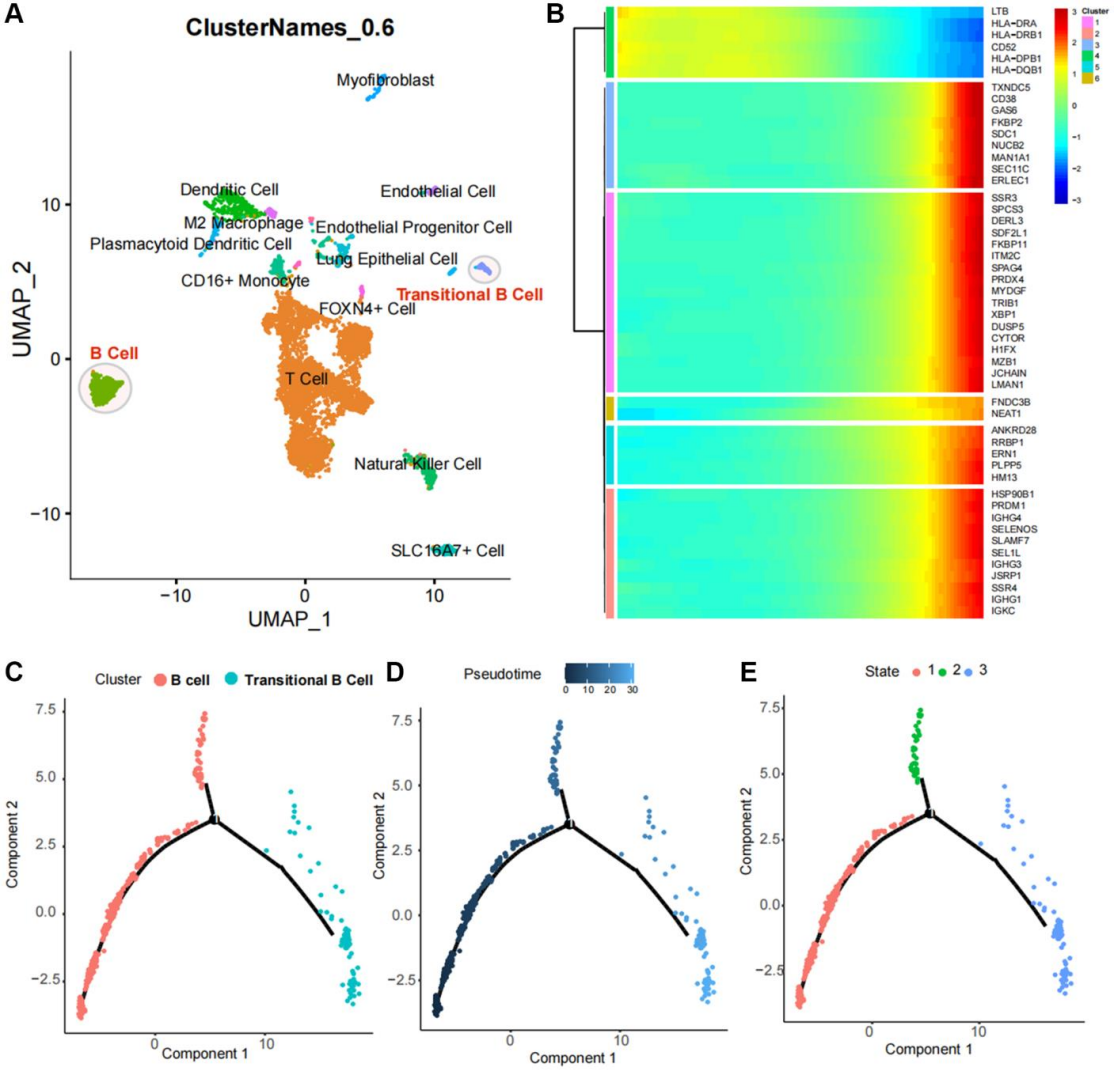
**Identification and pseudotime analysis of single-cell subpopulations in lung cancer.** (**A**) Identification of immune cell subpopulations in lung cancer tissues, with T cells as the major subpopulation and B cell populations with two subpopulations spaced apart in the UMAP spatial map, suggesting distinct biological functions. (**B**) Single-cell pseudotime analysis heat map of B-cell subpopulations. (**C**–**E**) Spatial distribution of B-cell populations and Transitional B cells in the pseudotime analysis atlas.

Following the exclusion of cells exhibiting abnormal gene numbers and ribosome ratio, cell populations were downscaled by PCA and tSNE. Subsequently, these populations were subjected to automated database annotation (Figure 4A). Notably, T cells emerged as the predominant cell population in LUAD. Intriguingly, Transitional B cells and B cells, despite belonging to the same B cell population, exhibited spatial separation within the cell population space, implying a potential difference in their biological functions. As for the overall cell population, the pseudotime analysis revealed a decrease in the expression levels of LTB, HLA-DRA, HLA-DRB1, CD52, HLA-DPB1 and HLA-DQB1 during the late stage of LUAD progression. In contrast, the expression levels of TXNDC5, CD38, GAS6, FKBP2 and SDC1 were increased in the late stage of LUAD progression, indicating potential distinct roles in the occurrence and progression of the cancer (Figure 4B).

### Cell identification of core subpopulations, pseudotime analysis and variance analysis

Based on the above analysis, a subpopulation analysis of Transitional B Cells and B cells was conducted. The pseudotime trajectory revealed the presence of two cell populations with a core regulatory node in cell development (Figure 4C), with Transitional B Cells being classified within the late developmental population (Figure 4D, 4E).

### Single cell GSVA and pathway enrichment analysis

**Figure 5:**
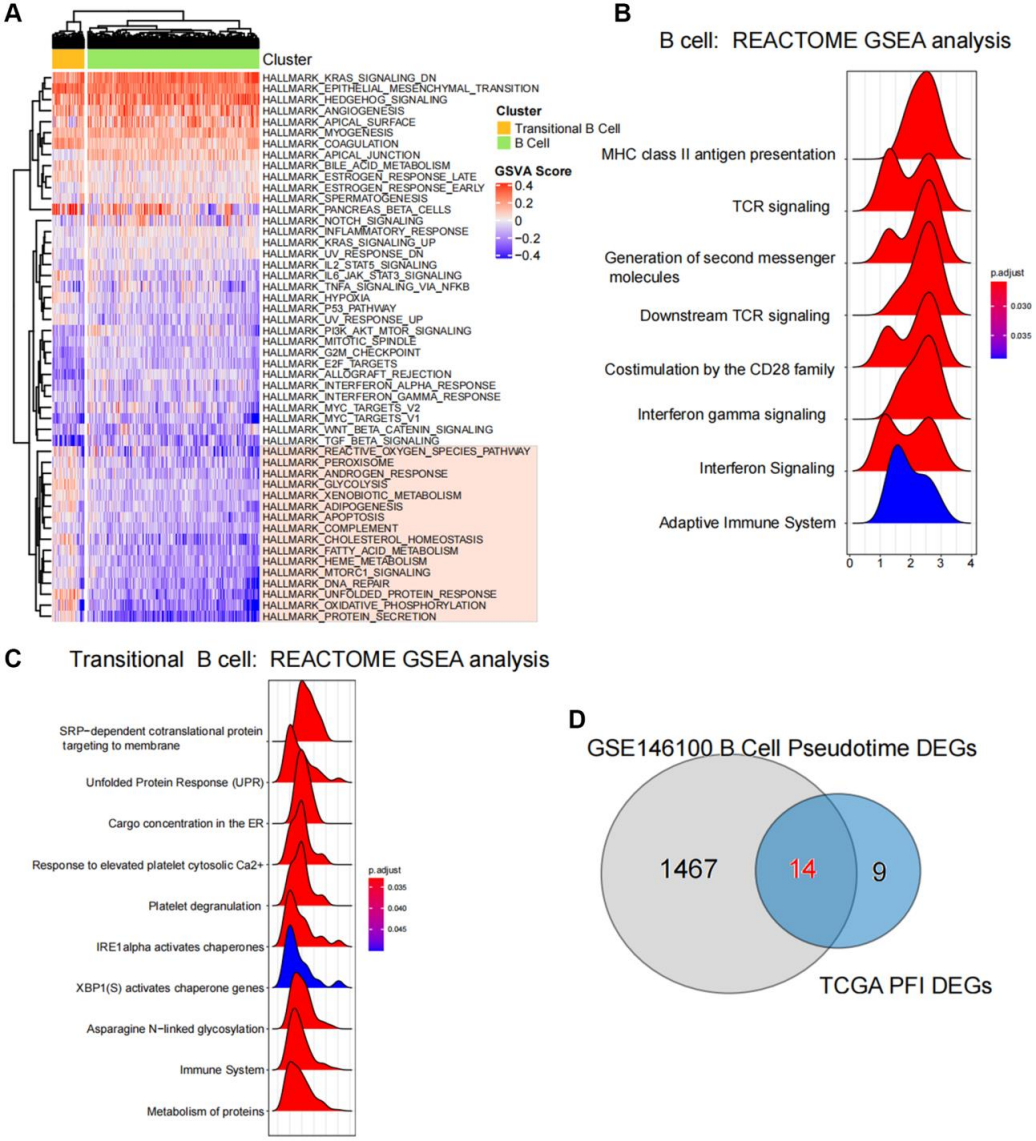
**Enrichment and variability analysis of B-cell subpopulation pathways.** (**A**) Variability scores of B cell subpopulations and Transitional B cells in the Hallmark gene set pathway. (**B**, **C**) Results of enrichment significance of B cell subpopulations and Transitional B cells at the REACTOME gene set level. (**D**) Intersection analysis of pseudotime regulatory genes of B-cell subpopulations and ferroptosis-related differential genes in TCGA.

We quantified the GSVA pathway in the expression profiles of cells from both populations, suggesting that HALLMARK DNA REPAIR HALLMARK UNFOLDED PROTEIN RESPONSE HALLMARK OXIDATIVE PHOSPHORYLATION and HALLMARK PROTEIN SECRETION were significantly elevated in the B cell population, while HALLMARK KRAS SIGNALING DN, HALLMARK EPITHELIAL MESENCHYMAL TRANSITION and HALLMARK HEDGEHOG SIGNALING demonstrated high expression in the transitional B cell population (Figure 5A).

Results of GSEA revealed a significant association between B cells and the activation of various pathways, including MHC class II antigen presentation, TCR signaling and Generation of second messenger molecules (Figure 5B), Additionally, the activation of Transitional B cells was closely related to processes such as SRP-dependent cotranslational protein targeting to membrane, the stressed Protein Response (UPR) and Cargo concentration in the stressed ER et al. (Figure 5C).

### Identification and functional analysis of core regulatory genes

**Figure 6:**
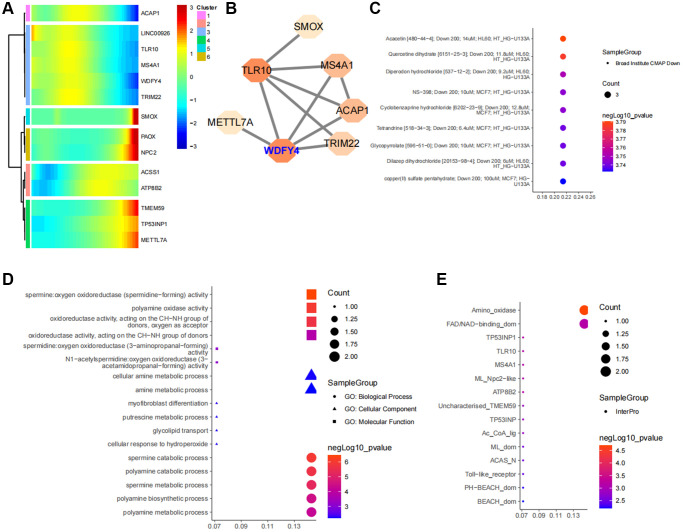
**Identification of ferroptosis-related target genes in B cell subpopulations of lung cancer.** (**A**) Developmental changes of common core genes in gene intersection of B cell pseudotime core genes and lung cancer ferroptosis differential genes in the B cell pseudotime heat map. (**B**) Network of intersection genes at the level of protein-protein interactions. (**C**) Enrichment analysis of intersection genes in CMAP drug database. (**D**) Enrichment analysis of intersection genes at the levels of Biological Process, Cellular Component and Molecular Function. (**E**) Predictive analysis of intersection genes in the InterPro database at levels of substructural interactions.

By acquiring intersections, we have identified 14 ferroptosis-related differential genes that are closely associated with B cell activation in LUAD (Figure 5D). Pseudotime analysis has suggested that the expression levels of ACAP1, LINC00926, TLR10, MS4A1, WDFY4 and TRIM22 decrease during at the later stages of B cell differentiation in LUAD, while the expression levels of TMEM59, TP53INP1 and METTL7A increase (Figure 6A). The PPI network highlights the significance of WDFY4 in the biological interaction network and its role as a crucial regulatory target for B cell differentiation in LUAD (Figure 6B). Drug target prediction indicated that Acacetin, Quercetine dihydrate and Diperodon hydrochloride were essential target drugs for WDFY4-mediated B cell differentiation in LUAD (Figure 6C). Furthermore, the spermine catabolic process, polyamine catabolic process and spermine metabolic process appear to significant pathways of WDFY4 (Figure 6D). Additionally, Amino_oxidase, FAD/NAD-binding_dom and TP53INP1 were identified as structural thresholds for target regulation (Figure 6E).

### Correlation between downregulation of WDFY4 and poor prognosis

**Figure 7:**
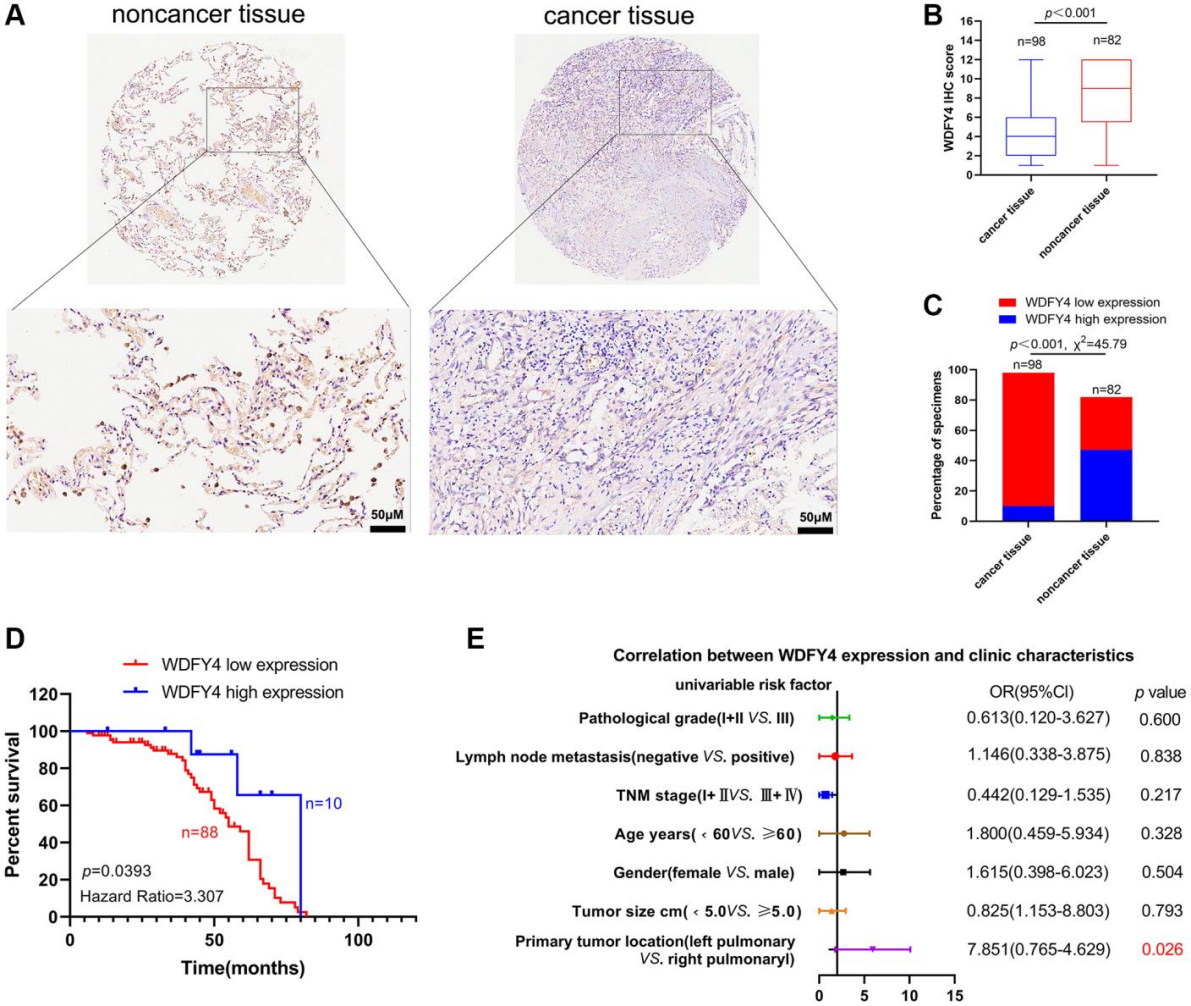
**The expression and clinical significance of WDFY4 in LUAD.** (**A**, **B**) Tissue microarray (TMA) analysis by IHC staining showed a low expression of WDFY4 in LUAD tissues. (**C**) High and low expression rates of WDFY4 in LUAD tissue. (**D**) Kaplan-Meier analysis showed that LUAD patients with downregulation of WDFY4 were positively correlated with worse prognosis and shorter overall survival. (**E**) Correlations of WDFY4 expression levels in LUAD tissues and clinicopathological features.

To conduct a more comprehensive examination of the expression of WDFY4 in LUAD tissues and its relationship with the prognosis of lung cancer patients, we analyzed the expression of WDFY4 in LUAD tissue microarray through immunohistochemistry (IHC). The findings revealed a lower expression level of WDFY4 in LUAD tissues compared to noncancerous tissues (Figure 7A, 7B), and a higher incidence of low WDFY4 expression in LUAD tissues compared to noncancerous tissues (Figure 7C). However, there was no significant correlation observed between the expression of WDFY4 and various clinical pathological features, including age, gender, tumor size. Descriptive grade, lymph node positivity, and TNM stage (Figure 7D, 7E, Table 1). Notably, Kaplan-Meier analysis demonstrated a consistent association between down-regulation of WDFY4 and a poorer prognosis (Figure 7D), indicating the potential oncogenic role and prognostic significance of WDFY4 in LUAD.

**Table 1:** Correlation of the expression of WDFY4 in lung cancer with clinicopathologic features.

**Characteristics**	**No. of patients**	**WDFY4**		***p*-value**
		**High expression *n* (%)**	**Low expression *n* (%)**	
Overall	98	10 (10.2)	88 (89.8)	
Age				
<60 years, *n* (%)	45 (45.9)	5 (11.1)	40 (88.9)	0.3280
≥60 years, *n* (%)	53 (54.1)	5 (9.4)	48 (90.6)	
Gender				
Female, *n* (%)	39 (39.8)	3 (7.7)	36 (92.3)	0.5042
Male, *n* (%)	59 (60.2)	7 (74.6)	52 (25.4)	
Tumor size				
<5.0 cm, *n* (%)	72 (73.5)	7 (9.7)	65 (90.3)	0.7931
≥5.0 cm, *n* (%)	26 (26.5)	3 (11.5)	23 (88.5)	
Primary tumor location				
Left pulmonary, *n* (%)	56 (57.1)	9 (16.1)	47 (83.9)	0.0267
Right pulmonary, *n* (%)	42 (42.9)	1 (2.4)	41 (97.6)	
Pathological grade				
I + II, *n* (%)	69 (70.4)	3 (4.3)	66 (95.7)	0.6007
III, *n* (%)	29 (29.6)	2 (6.9)	27 (93.1)	
Lymph node metastasis				
Negative, *n* (%)	52 (53.1)	5 (9.6)	47 (90.4)	0.8378
Positive, *n* (%)	46 (46.9)	5 (10.9)	41 (89.1)	
TNM stage				
I + II, *n* (%)	66 (67.3)	5 (7.6)	61 (92.4)	0.217
III + IV, *n* (%)	32 (32.7)	5 (15.6)	27 (84.4)	

### Overexpression of WDFY4 inhibits the proliferation and migration of A549 Cells and promotes apoptosis *in vitro* and *in vivo*

**Figure 8:**
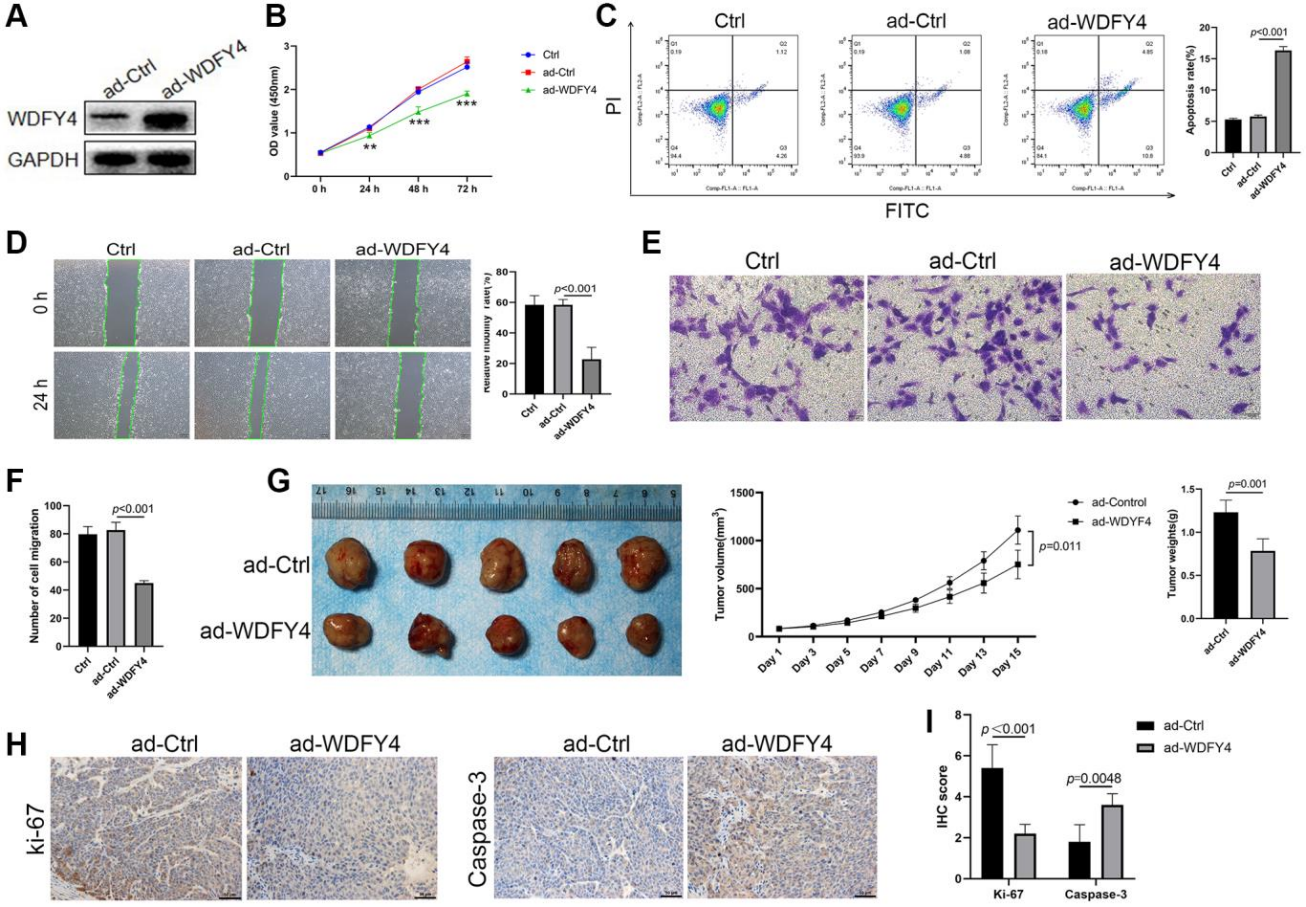
**The effect of WDFY4 on the biological characteristics of lung adenocarcinoma A549 cells *in vitro* and *in vivo*.** (**A**) The expression level of WDFY4 in A549 lung adenocarcinoma cells transfected with ad-Ctrl and ad-WDFY4. (**B**) Cell viability was measured by CCK-8 assays. (**C**) Cells were collected and stained with PI and annexin V-FITC for apoptosis analysis by flow cytometry. (**D**–**F**) Scratch wound healing and Transwell migration assay analyzed the metastasis of A549 cells after transfection of ad-WDFY4. (**G**) A549 cells transfected with ad-Ctrl and ad-WDFY4 were transplanted subcutaneously into nude mice, and the cancer growth curve and cancer weight were monitored (*n* = 5). (**H**, **I**) The expression levels of Ki-67 and Caspase-3 in cancer tissues were analyzed by immunohistochemistry (*n* = 5).

To investigate the biological effect of WDFY4 on lung adenocarcinoma cells, we employed the lung adenocarcinoma A549 cell line and used lentivirus to transfect A549 cells to establish a WDFY4 overexpression cell model. Our findings demonstrate that the transfection of ad-WDFY4 significantly increased the expression of WDFY4 in A549 cells (Figure 8A). Moreover, the overexpression of WDFY4 inhibited the proliferation of A549 cells (Figure 8B), as evidenced by a notable increase in the apoptosis rate within the ad-WDFY4 group compared to the ad-Ctrl group (Figure 8C). Furthermore, we employed the Scratch wound healing assay and Transwell migration assay to investigate the effect of WDFY4 on the metastatic potential of the A549 cells. The results depicted in Figure 8D–8F demonstrate that the upregulation of WDFY4 substantially inhibits the migratory capacity of the A549 lung adenocarcinoma cell line. To investigate the potential influence of WDFY4 on the progression of lung cancer *in vivo*, we subcutaneously injected A549 cells that were stably expressing WDFY4 following lentivirus infection into nude mice. The analysis of subcutaneous tumor formation revealed a significant decrease in tumor volume and weight of nude mice in the ad-WDFY4 group compared to those in the ad-Ctrl group. Furthermore, immunohistochemical analysis demonstrated a lower expression level of the proliferation-related antigen ki-67 in the ad-WDFY4 group compared to the ad-Ctrl group, while the expression level of the apoptosis-related protein Caspase-3 was higher in the ad-Ctrl group (Figure 8G–8I). The data presented herein indicate that the upregulation of WDFY4 exerts a substantial inhibitory effect on the proliferation of lung adenocarcinoma cells both *in vitro* and *in vivo*, thereby highlighting its potential as a viable therapeutic target for the treatment of lung adenocarcinoma.

## DISCUSSION

Non-small cell lung cancer (NSCLC) is predominantly diagnosed in its advanced stage, rendering conventional treatments ineffective. Statistics suggest that the 5-year survival rate for stage IB NSCLC patients stands at 68%, whereas patients in stages IVA-IVB exhibit a survival rate ranging from 0–10% [23]. Consequently, there is an urgent need to explore novel treatment approaches to enhance the survival outcomes for individuals afflicted with lung cancer. In this context, ferroptosis, an emerging form of cell death, exhibits a close association with the progression and management of lung cancer. Previous studies have demonstrated the significant upregulation of NFS-1 in lung cancer tissues and its ability to effectively mitigate hyperoxia-induced ferroptosis in lung cancer cells, its potential as a protective agent against ferroptosis in this context [24]. Additionally, the RNA-binding protein RBMS1, known for its binding affinity to the c-Myc gene, has been found to exert transcriptional repression on target genes. In the case of lung cancer cells, RBMS1 inhibits SLC7A11 expression, thereby reducing SLC7A11-mediated cystine uptake and facilitating ferroptosis in lung cancer cells [25]. The loss of function and mutation of STK11 and KEAP1 in LUAD have been found to be associated with cell invasion, proliferation, and resistance to chemotherapy. STK11/KEAP1 co-mutations lead to a notable increase in the expression of ferroptosis-protective genes, including SCD and AKR1C1/2/3, as well as resistance to drug-induced ferroptosis in lung cancer cells [26]. These findings highlight the potential of cellular ferroptosis as a novel treatment for NSCLC, with multiple targets capable of inducing ferroptosis. In the present study, we found that FPI values in LUAD tumor tissues were lower than those in para-cancerous tissues through the application of GSAV and FPI analysis. Furthermore, a correlation was established between lower FPI values and reduced long-term survival rates among LUAD patients. These findings align with previous research indicating that lung cancer cells exhibit specific resistance to ferroptosis. Consequently, the facilitation of ferroptosis in lung cancer cells holds potential benefits for the treatment of lung cancer.

The correlation between the immune status and immune cell infiltration in NSCLC and the level of ferroptosis in tumor cells is well-established. Several researchers have demonstrated that the construction of a prognostic model using ferroptosis-related genes reveals an association between ferroptosis-related risk scores and immune status in lung cancer [27]. While the role of T cells in tumor progression has been extensively studied, but the function of B cells in this context remains uncertain. A study has indicated that B cells are key components of immune cell infiltration in various solid tumors, including breast cancer and melanoma, constituting nearly half of all infiltrating lymphocytes [28]. Similarly, lung cancer has also been found to harbor infiltrating B cells, with their phenotype varying depending on clinical stages and histological subtypes [29]. These B cells are associated with a dual function of both promoting and inhibiting tumor growth [28]. Recently, it has been discovered the Burkitt-like stage of B cell development requires the detoxification of lipid ROS by glutathione peroxidase 4 (GPX4) and its cofactor glutathione to inhibit cellular ferroptosis. Also, GPX4 is necessary for the prevention of ferroptosis during the development, maintenance, and response of innate-like B cells [30, 31]. Our investigation revealed a significant correlation between the infiltration levels of various immune cells, including B cells, CD8. T. cells, T. helper. cells, Th17.cells, NK.CD56bright.cells and NK.CD56dim.cells, and the occurrence of ferroptosis in LUAD, with B cells exhibiting the strongest association. Subsequent GSEA analysis revealed a significant upregulation of the pathways related to B cell activation, B cell proliferation, B cell-mediated immunity and B cell receptor signaling pathway in the PFI low-score group. Conversely, these pathways were expressed at lower levels in the PFI high-score group. These findings indicate a strong association between ferroptosis and B cell activation, corroborating the previously reported results that highlight the regulatory role of ferroptosis in B cell infiltration and differentiation. Furthermore, our results indicate a strong correlation between B cell infiltration and the long-term survival prognosis of patients, thereby proposing the induction of ferroptosis in tumor cells as a potential mechanism to enhance B cell infiltration and activation for immunotherapy in lung cancer.

To further explore the infiltration of B cells in lung cancer, scRNA-seq data of LUAD were analyzed, revealing that B cells constitute a significant immune cell population alongside T cells, encompassing Transitional B cells, which represent a cell population in advanced stages of development. B cells are closely related to the activation of MHC class II antigen presentation, TCR signaling and the generation of second messenger molecules. Additionally, SRP-dependent cotranslational protein targeting to the membrane, Unfolded Protein Response (UPR), and Cargo concentration in the ER are closely associated with the activation of Transitional B cells. The presence of these findings confirms the infiltration of B-cell populations in LUAD tumor tissue, thereby offering valuable insights for immune therapy and clinical application.

As the fourth member of the WDFY family, WDFY4 exhibits high expression levels in various immune tissues such as lymph nodes, spleen, thymus and tonsils. However, the specific functions of WDFY4 remain poorly understood [32]. Several studies have demonstrated a correlation between WDFY4 and certain autoimmune diseases, such as systemic lupus erythematosus, rheumatoid arthritis, juvenile idiopathic arthritis and clinical anaplastic dermatomyositis [33, 34]. Recent findings have indicated that WDFY4 plays a crucial role in regulating cDC1-mediated cross-presentation of viral and tumor antigens. The cDC1 cells in WDFY4 mice failed to control tumor growth, trigger viral-specific CD8+ T cells, or induce tumor rejection *in vivo* [34]. The overall survival rate is longer in melanoma patients with high WDFY4 expression, which is highly correlated with the progression of cancer and autoimmune disorders [35]. In this study, we identified and functionally analyzed core regulatory genes to determine their association with B cell activation in LUAD. Through this analysis, we obtained 14 ferroptosis-related differential genes that are closely linked to B cell activation in the context of lung cancer infiltration. Pseudotime analysis showed a decrease in the expression levels of ACAP1, LINC00926, TLR10, MS4A1, WDFY4 and TRIM22 during the late stage of LUAD B cell differentiation, while the expression levels of TMEM59, TP53INP1 and METTL7A were found to be increased. The PPI network indicated that WDFY4 was important in the biological interaction network, identifying it as a crucial regulatory target for LUAD B cell differentiation. Furthermore, previous research has reported the involvement of WDFY4 in the progression of SLE symptoms through the regulation of B cells via a non-classical autophagic pathway [34]. To further explore the relationship between WDFY4 and lung cancer, an immunohistochemical analysis of LUAD tissue microarray was conducted. The results indicated that WDFY4 was notably reduced in LUAD tissues, and the patients with low WDFY4 expression exhibited a worse prognosis, particularly in relation to the primary sites of lung cancer. Subsequently, drug target prediction was conducted, revealing Acacetin, Quercetine dihydrate and Diperodon hydrochloride as essential target drugs for WDFY4-mediated B cell differentiation in LUAD. Simultaneously, we employed lentivirus transfection as a means to establish a cell model with overexpressed WDFY4, and found that the overexpression of WDFY4 could inhibit the growth of lung adenocarcinoma A549 cells both *in vitro* and *in vivo*.

Nonetheless, it is imperative to acknowledge the limitations of our study and address the areas that require further refinement. Firstly, it is imperative to conduct an analysis to determine the potential association between the infiltration and differentiation levels of B cell population in LUAD and the overall survival of patients. Secondly, it is crucial to elucidate the relationship between differentiation and infiltration of B cells and ferroptosis. Thirdly, a comprehensive examination should be undertaken to evaluate the differentiation and infiltration of B cells by expressing or knocking down WDFY4, with the ultimate aim of exploring the potential mechanism of WDFY4 as targeted immunotherapy in lung cancer by regulating B cells. Finally, it is imperative to investigate the indispensability of Acacetin, Quercetine dihydrate and Diperodon hydrochloride as target drugs for WDFY4-mediated B cell differentiation in LUAD.

## CONCLUSIONS

In the current investigation, we have confirmed a robust correlation between ferroptosis and the prognosis of patients with LUAD. Promoting ferroptosis in LUAD cells induces infiltration and differentiation of B cells, thereby presenting a new direction for LUAD immunotherapy. In addition, our analysis of single-cell data analysis LUAD has confirmed the significance of the B cell population as a crucial constituent of immune cell infiltration in this context. Furthermore, we have identified WDFY4 as a pivotal gene responsible for regulating the infiltration and activation of B cells. Moreover, it has been observed that LUAD patients with low WDFY4 expression exhibit a more unfavorable prognosis. Overexpression of WDFY4 has shown to inhibit the growth of lung adenocarcinoma A549 cells both *in vitro* and *in vivo*. These findings suggest that WDFY4 holds significant potential as a target for immunotherapeutic interventions in lung cancer.
